# Finding the Hidden Risk Profiles of the United States Opioid Epidemic: Using a Person-Centered Approach on a National Dataset of Noninstitutionalized Adults Reporting Opioid Misuse

**DOI:** 10.3390/ijerph17124321

**Published:** 2020-06-17

**Authors:** Francisco A. Montiel Ishino, Tamika Gilreath, Faustine Williams

**Affiliations:** 1 Division of Intramural Research, National Institute on Minority Health and Health Disparities, National Institutes of Health, 7201 Wisconsin Ave, Ste.533G6, Bethesda, Rockville, MD 20814, USA; faustine.williams@nih.gov; 2 Transdisciplinary Center for Health Equity Research, Department of Health Education, College of Education and Human Development, Texas A&M University, 4243 TAMU, 311F Blocker Building, College Station, TX 77843, USA; tgilreath@tamu.edu

**Keywords:** latent class analysis, analgesics, opioid, social determinants of health, person-centered approach

## Abstract

Opioid misuse can lead to use disorder and other adverse outcomes. Identifying sociodemographic risk profiles and understanding misuse patterns in combination with health indicators can inform prevention science and clinical practice. A latent class analysis of opioid misuse was conducted on noninstitutionalized United States civilians aged 18 and older that reported opioid dependence or abuse in the 2017 National Survey of Drug Use and Health (*n* = 476; weighted *n* = 2,018,922). Opioid misuse was based on heroin and/or prescription pain reliever use, and associated determinants of health and mental health indicators. Five misuse profiles were identified: (1) single heroin or prescription misuse with high-income; (2) female prescription pain reliever misuse with psychological distress and suicidality; (3) younger polyopioid misuse with the highest proportion of Hispanics and heroin use; (4) older polyopioid misuse with the highest proportion of non-Hispanic blacks and disability; and (5) older non-Hispanic white male exclusive dual heroin and/or prescription misuse (27%, 20%, 38%, 10%, and 5% of sample, respectively). The identified risk profiles can inform public health practice to develop interventions for acute and immediate response by providing etiological evidence and to inform prevention and intervention efforts along the continuum from opioid initiation to use disorder.

## 1. Introduction

The statistics demarcating the opioid epidemic in the United States (US) are staggering and sobering. Opioid misuse has resulted in an estimated 1.7 million years of life lost in 2016 [1], and this number is projected to increase in the coming years. In 2017, more than 67.8% (47,600) of the 70,237 overdose deaths reported involved opioids; compared to the almost 42,000 reported in 2016 [2,3]. Although the true extent of overdose deaths are not known [4], it is reported that over 130 Americans die daily from an opioid related overdose [5]. Chen et al. [6], used a computer simulation model to analyze data from the National Survey on Drug Use and Health from 2002 to 2015 and projected that deaths due to illicit opioids to increase by 61% by 2025.

Factors such as opioid misuse and mortality contributing to the ongoing crisis are complex and not well understood. Some researchers have attributed the overprescribing of opioids as a leading cause to use disorder and addiction [4,6,7,8]. Current preventive intervention programs have targeted overprescribing, as well as multiple other factors to stem opioid misuse and overdose. These prevention and intervention strategies/programs have been implemented in clinical and community settings to curb the epidemic. Clinical interventions include prescription drug monitoring programs, identifying individuals seeking opioids inappropriately, psychosocial counseling, and medication-assisted treatment [9]. Community setting interventions include needle exchange programs as well as referral services to clinical treatment, public health services, and recovery programs [9]. However, these interventions, have not been fully effective in curtailing the opioid crisis. Furthermore, a 5-year projection study found that overdose deaths could increase through heroin use due to prescription restriction interventions among patients [10].

Various factors such as age, race/ethnicity, county population levels, and social determinants of health are related to opioid misuse and overdose deaths [2]. Evidence suggests that prescribed opioids for chronic pain misuse among patients is between 21 and 29% in the US, and 12% of those patients develop a use disorder [3,5]. Furthermore, multiple determinants of health—such as demographic characteristics [11,12], socioecological indicators [13], overall general and mental health status [14,15], and co-substance use [16,17]—have been related to misuse and opioid use disorders [18,19]. Generally, studies utilize regressions to determine how co-variates increase or decrease risk of opioid misuse or addiction. The literature is clear that multiple determinants of opioid misuse and use disorder co-occur [20] and understanding the patterns of co-occurrence may aid in prevention planning, clinical screening, and provide data to improve targeting and tailoring of interventions. Given there is need to prevent prescription misuse and heroin use to use disorder, our purpose was to identify profiles of risk using social determinants of health and mental health indicators associated with opioid use, as well as examine correlates of prescription drug and heroin co-use.

To ameliorate the public health burden and reduce opioid-related deaths, a need exists to identify opioid misuse risk profiles. We used latent class analysis to identify profiles of opioid misuse by heroin use, prescription pain reliever use, or heroin and prescription pain reliever use with co-occurring determinants of health, including mental health and other substance use. This study is among the first to use a person-centered approach, to identify opioid misuse risk profiles. While it is known that opioid misuse includes heroin, prescription pain relievers, or a combination of both, the respective misuse risk profiles are unknown. Our study fills a critical gap in the literature by identifying opioid misuse risk profiles to better inform prevention strategies, provide guidance for clinical screening, and tailoring targeted intervention programs.

## 2. Materials and Methods

The current study used the 2017 National Survey on Drug Use and Health (NSDUH), which provided a representative sample of noninstitutionalized US adults 18 years and older that self-reported opioid dependence or abuse in all 50 states and District of Columbia. The NSDUH uses multistage probability sampling to estimate substance use and mental health at the national to substate level among the US noninstitutionalized population using audio computer-assisted self-interviews [21]. The Institutional Review Board assessed the research protocol, and determined no approval was necessary as no human participants were involved in this study. Further detail on the NSDUH sampling strategy and design, in addition to the data analyzed in this study were from the 2017 NSDUH cycle and are publicly available from the Substance Abuse & Mental Health Data Archive repository at the following link: https://www.datafiles.samhsa.gov/study/national-survey-drug-use-and-health-nsduh-2017-nid17938.

Opioid misuse profiles were identified and named using the following 14 observed variables from the 2017 NSDUH: (1) age group (i.e., 18–25; 26–34; 35–49; and 50 and older); (2) sex/gender (i.e., male and female); (3) race/ethnicity (i.e., non-Hispanic white; non-Hispanic black; Hispanic; and Other racial/ethnic group that included Native American/Alaska Native, Native Hawaiian/other Pacific Islander, and Asian); (4) sexual identity (i.e., heterosexual and sexual minorities such as lesbian, gay, and bisexual); (5) residence based on core-based statistical areas or CBSAs [21] of 1 million or more, less than 1 million, or not in a CBSA as defined by the Office of Management and Budget [22]; (6) family income (i.e., less than $20,000; $20,000 to $49,000; and $50,000 or more); (7) educational attainment (i.e., less than high school; high school graduate; some college/associate’s degree; and college graduate); (8) employment in past week (i.e., employed full- or part-time; unemployed; disabled; and other category that included keeping house, in-school/training, retired, or does not have a job for other reason); (9) past criminality (i.e., arrested and booked in lifetime); (10) self-reported health (i.e., fair/poor; good; and very good/excellent); (11) private health insurance; (12) serious psychological distress in past year; (13) suicidality in past year (i.e., seriously thought about killing yourself); and (14) type of opioid dependence or abuse (i.e., heroin, prescription pain reliever, or both heroin and prescription pain reliever). Substance dependence or abuse of substances other than opioid were accounted for as covariates.

### 2.1. Other Substance Dependence or Abuse Covariates

Nicotine dependence in the past month was assessed using Nicotine Dependence Syndrome Scale scores, as well as the Fagerstrom Test of Nicotine Dependence scale. Self-reported alcohol and marijuana dependence or abuse in the last year was also ascertained. Dependence or abuse in the past year were ascertained for the following illicit substances: cocaine, hallucinogens, inhalants, methamphetamines, tranquilizers, stimulants (independent of methamphetamine), and sedatives.

### 2.2. Latent Class Analysis Model Selection Criteria

All analyses accounted for the 2017 NSDUH complex survey design to best provide a representative sample of noninstitutionalized civilian US adults. All models were weighted and accounted for clustering and stratification. A model comparison approach was used to determine the number of classes. Multiple models were created (i.e., 1-, 2-, 3-class solutions, etc.) to then select the best model based on two criteria: (1) High entropy (i.e., the acceptable quality of classification); and (2) sample-size-adjusted Bayesian information criterion (ssaBIC) [23]. Models were also assessed on their practical and theoretical implications. The 1-class solution model was calculated to assess fit indices and compare with subsequent models. Covariates were assessed on the model selected for interpretation using multinomial logistic regression. All LCAs were conducted using Mplus 8.2 (Muthén & Muthén).

## 3. Results

The sample selected for analysis from the 2017 NSDUH was restricted to noninstitutionalized US adults 18 years and older who self-reported opioid dependence or abuse (*n* = 476; weighted *n* = 2,018,922). Age groups were almost evenly distributed between in the sample. The weighted sample was mostly male (60.6%), non-Hispanic white (77.8%), resided in a CBSA with less than 1 million people (48.4%), heterosexual (90.5%), had a family income of $50,000 or more (39.8%), had some college or an associate’s degree (39.2%), employed (53.3%), past criminality (61.8%), self-reported good health (37.1%), had serious psychological distress (56.2%), reported no suicidality (71.9%), and was not covered by private health insurance (64.4%).

The majority of the weighted sample used only pain relievers (66.0%). The weighted sample also reported 55.6% nicotine dependence in the past month, as well as 24.7% alcohol and 17.6% marijuana dependence or abuse in the past year. Other concurrent illicit substance dependence or abuse in the past year (35.2%) was also reported. See Table 1 for full sample descriptive characteristics.

**Figure 1:**
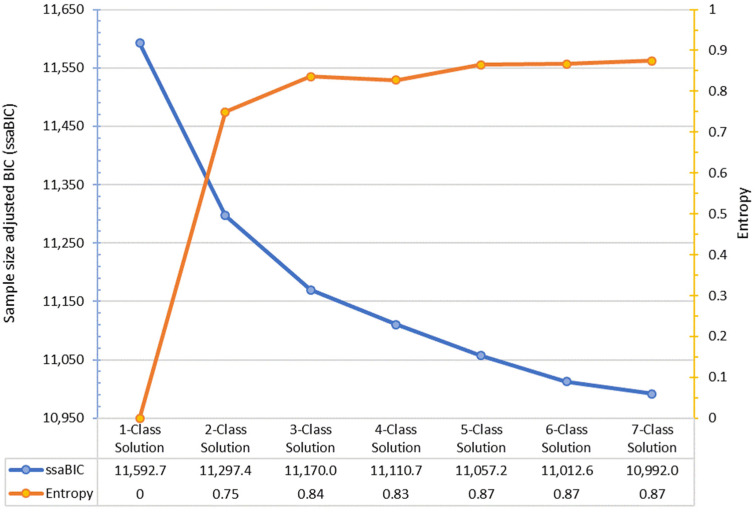
Model fit information criteria of latent classes compared for analysis.

Our opioid misuse profiles were identified using LCA. The best model fit selected was a five-class solution that had a low ssaBIC (11,057.2) and high entropy. The entropy or classification accuracy of the selected model was 0.87, which indicated a reliable separation of profiles or classes (see Figure 1). Profiles were named after highest likelihood of opioid(s) misused, as well as sociodemographic indicators (i.e., sex and age group). Opioid misuse categorization was based on single, dual, or poly use type.

Class 1, or single heroin or prescription misuse profile (27.3% of sample), misused prescription pain relievers or heroin (78.5% and 14.9% conditional probability, respectively). This profile was not defined by a single age group but had the highest conditional probability of being 50 and older (35.1%). The single opioid misuse profile had a high likelihood of being male (63.0%), non-Hispanic white (78.1%), identifying as heterosexual (95.3%), reporting a family income of more than $50,000 (72.2%), had some college or an associate’s degree (46.0%), and employed (92.7%), as well as did not report serious psychological distress (60.4%) or suicidality (78.0%). Class 1 exclusively had private health insurance, and had the highest conditional probability when compared to any other class. This class self-reported a higher likelihood of good (44.8%) and very good/excellent health (47.2%).

Class 2, or female prescription pain reliever misuse profile (20.3% of sample), primarily misused prescription pain relievers followed by combined heroin and prescription pain reliever use (83.5% and 12.5% conditional probability, respectively). This class had a higher likelihood of being 18 to 25 (32.4%) and 26 to 34 years old (31.3%). This dual misuse subgroup was found to have the highest conditional probabilities of being female (77.9%), identifying as a sexual minority (22.1%), some college or an associate’s degree (50.0%), other employment classification (30.1%), not arrested (67.2%), and serious psychological distress (89.6%). Class 2 had a high likelihood of making $20,000 to $49,999 (40.4%), residing in CBSAs with less than 1 million (52.8%), with private health insurance (34.0%), reporting serious psychological distress within the past year (76.7%) and suicidality (53.2%).

Class 3, or younger polyopioid misuse profile (37.5% of sample), reported heroin, prescription pain relievers, or a combination of heroin and prescription pain relievers (37.1%, 48.3%, and 14.6% conditional probability, respectively). This class was younger as they had a higher likelihood of being under the age of 50. The profile had a higher conditional probability of being 26–34 years of age (42.4%), male (71.0%), non-Hispanic white (76.3%), heterosexual (93.8%), family income less than $20,000 (39.5%), arrested and booked (83.2%), self-reported good health (43.0%), and no private health insurance (95.2%). This profile reported the highest conditional probabilities of being Hispanics (9.6%), a high school graduate (45.6%), unemployed (27.4%), residing in a CBSA with less than 1 million (53.8%), and no suicidality (83.9%). This polyopioid class had the highest conditional probability of heroin use compared to all profiles.

Class 4, or older polyopioid misuse profile (10.1% of sample), reported use of heroin, prescription pain relievers, or a combination of heroin and prescription pain relievers (28.6%, 55.8%, and 15.6% conditional probability, respectively). The older polyopioid profile had a high conditional probability of being 50 and older (76.9%), male (79.0%), heterosexual (92.6%), and in serious psychological distress (71.9%). This polyopioid profile had the highest conditional probabilities of being non-Hispanic black (23.6%), reporting a family income less than $20,000 (52.9%), disability (79.7%), arrested and booked (91.8%), self-reported fair/poor health (100%), and no private health insurance (100%).

**Figure 2:**
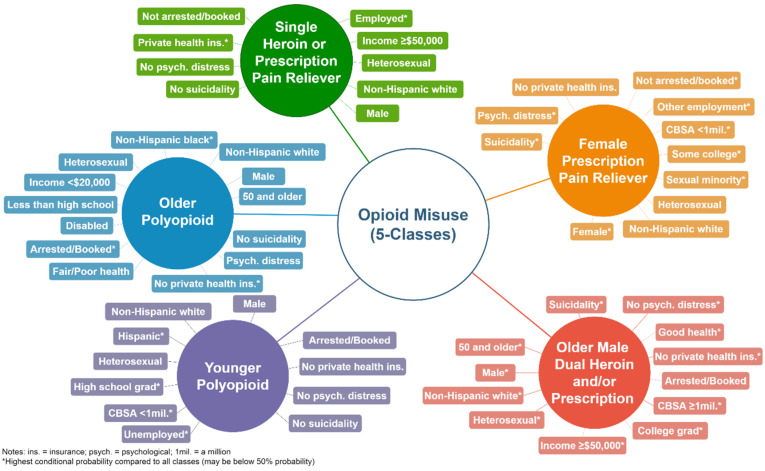
Opioid misuse latent classes with highest conditional probabilities within and between profiles. Within profile conditional probabilities are shown if above 50%.

Class 5, or older male dual heroin and/or prescription misuse profile (4.7% of sample), was found to misuse either prescription pain relivers or a combination of heroin and prescription opioids (78.6% and 21.4% conditional probability, respectively). This class was exclusively 50 years and older, male, non-Hispanic white, heterosexual, resided in a CBSA with more than 1 million individuals, self-reported good health, and had no private health insurance. This class had a high likelihood of having a family income of $50,000 or more (77.3%), being a college graduate (54.7%), disabled (44.0%), been arrested and booked (65.8%), and no serious psychological distress (66.7%). This dual misuse group had the highest conditional probability of combined heroin and prescription pain reliver use. See Table 2 for all conditional probabilities. Figure 2 illustrates the finding of the latent class model.

Our covariate analysis using multinomial logistic regression of other substance dependence or abuse revealed the following. First, that the younger polyopioid misuse profile (Class 3) was over 350% more likely to report nicotine dependence in the last month (odds ratio (OR) = 4.49, 95% confidence interval (CI): 1.94–10.4), and was almost 78% less likely to report marijuana dependence or abuse (OR = 0.22, 95% CI: 0.08–0.60) compared to the female prescription misuse profile (Class 2). The older polyopioid misuse profile (Class 4) was over 270% more likely to report nicotine dependence in the last month (OR = 3.74, 95% CI: 1.00–14.0) than the female prescription misuse profile (Class 2). See Table 3 for more detail.

## 4. Discussion

Our study revealed five classes of opioid misuse and their respective risk profiles based on sociodemographic characteristics and type of opioids used: (1) A single heroin or prescription misuse class with high probabilities of high-income, employment, and private health insurance; (2) a female prescription pain reliever misuse class with some combination use and high probabilities of serious psychological distress and suicidality; (3) a younger polyopioid misuse class with the highest likelihood of heroin use and being Hispanic; (4) an older polyopioid misuse class with highest probabilities of being non-Hispanic black and disabled with the second highest conditional probabilities of heroin and combined heroin and prescription pain reliever use; and (5) an older non-Hispanic white male exclusive dual heroin and/or prescription misuse class with high-income and no private health insurance residing in high population density areas.

Sociodemographic indicators were useful in identifying each profile, as well as possible disparities and points of comparison between profiles. However, participants of color, were limited in our opioid misuse sample. Nevertheless, non-Hispanic blacks had the highest likelihood of being identified with the older polyopioid misuse group. Hispanics had the largest ethnic representation among the younger polyopioid misuse group. Single heroin or prescription misuse and female prescription pain reliever misuse profiles were predominantly non-Hispanic white. The dual older male heroin and/or prescription use class was the only exclusive non-Hispanic white profile. While it may seem that the US media has portrayed the risk profiles of non-Hispanic male whites like those of the dual heroin and/or prescription users, the opioid epidemic involves many racial/ethnic groups in addition to other demographic characteristics [24]. For instance, while the conditional probabilities of non-Hispanic black and Hispanic were relatively small in our study, Scholl et al. [2] reported that they had the largest increase in opioid overdose death compared to other racial ethnic groups. Furthermore, we assessed our risk profiles based on the likelihood of combined heroin and/or prescription opioid use.

We considered the dual opioid and/or prescription opioid misuse profile to be the highest at-risk group because of the reported likelihood of a combination of heroin and prescription pain reliever use. The combination opioid misuse suggests that this class may have transitioned to heroin use from prescription pain relievers since there was no likelihood of heroin only use. This class exclusively used prescription opioids that are known to be risk factor to heroin use [14,25,26,27,28]. Becker et al. [14], using the 2001–2004 NSDUH, found that individuals using heroin had almost four times increased odds of reporting nonmedical use of opioids, as well as almost triple the odds of being dependent on or abusing opioids compared to those who did not use heroin. Similarly, Muhuri et al. [28], used multiple waves of the NSDUH, and found that approximately 80% of recent heroin users had initiated opioid use with nonmedically prescribed pain relievers. Further, Jones [27] analyzed the US epidemiological data, and found that over 77% of combination heroin and nonmedical prescription opioid users reported opioid initiation with prescription pain relievers.

A temporal effect may also exist with combination opioid use and initiation. Cicero et al. [26] reported that from the 1960s to the 1990s there has been a near linear decrease in heroin being the opioid initiation. Based on the prescription or combination opioid use class being exclusively age 50 and older, if opioid initiation were to have occurred between the 1960s and 1970s, the retrospective probability of heroin use would have decreased from 80% to 70%, respectively. Inversely, from the 1990s to the 2000s, there was a 50% to 75% probability of opioid misuse due to prescription pain relievers. In the 2010s, there was another shift where heroin initiation began to increase as prescription opioid initiation dropped [26]. Initiation cannot be ascertained or if a transition occurred; nevertheless, the high combination use of opioids within this class is indicative of specialized prevention strategies. Particularly as past-year prescription opioid misuse has been related to a lower perception of harm from heroin initiation and risk of regular use [29].

The older polyopioid misuse class had the second highest likelihood of combined heroin and prescription opioid use, as well as the second highest likelihood of heroin-only use, followed by the younger polyopioid misuse profile. The younger polyopioid profile had the highest conditional probability of heroin-only use. While these profiles may be affected by temporality and initiation effects of heroin use, as previously discussed, combination opioid and heroin-only use have been associated with various other factors. Factors include geographic residence (i.e., rural versus urban), socioeconomic status, socioecological factors (e.g., criminality), sexual identity (i.e., sexual minority), overall health (i.e., poor/fair health), mental health issues (e.g., psychological distress, depression, or anxiety), suicidality, and substance-dependence/abuse [13,14,15,18,19]. Furthermore, polyopioid users have been associated with moving from the intended oral administration route of prescription pain relievers to non-oral routes of administration.

Polyopioid misuse may take routes of ingestion via non-intended forms (e.g., chewing; mixing with water or other substances; rectal administration), inhalation (e.g., smoking, snorting, or vaping), or injection [30,31,32]. Using alternative routes of administration can lead to faster opioid tolerance that may necessitate a new and preferred dosing route to increase the potency of the narcotic effect resulting in patterns of increasing abuse [30,31,32]. Other bodily harms are also associated with alternative routes of administration that can range from minor irritations to tissue necrosis. Intravenous routes of administration have also been associated with increased risk of HIV and hepatitis C viral exposure [33,34,35]. The ultimate consequence of alternative routes of administration are unintentional death due to overdose [30,31].

Concurrent substance dependence and abuse has been positively associated with opioid misuse [16,36], moreover nicotine dependence has been linked to increased opioid use [17,37]. Among the identified profiles, we found that nicotine dependence was found to be at increased odds in both the younger and older polyopioid misuse profiles when compared to the female prescription misuse profile. The younger polyopioid misuse group was found to be at decreased odds of marijuana dependence and abuse when compared to the female prescription misuse profile. Marijuana has a mixed relationship with opioid misuse, and has been associated with increased and decreased use [38].

The current opioid crisis is unlike prior substance use crises of the past. It is important to develop interventions for acute and immediate response, but it is equally important to conduct research that provides etiological evidence and findings that can be used to inform prevention and intervention efforts along the continuum from opioid initiation to use disorder. The present work sought to add to the body of literature related to the opioid epidemic by identifying risk profiles of misuse among noninstitutionalized adults, which can further help inform prevention science and public health research. While existing studies on opioid misuse and use disorder exist, we took this into consideration when developing our person-centered methodological approach. We used the existing literature to inform our selection of opioid misuse indicators to identify how each one played a role within opioid misuse profiles as well as to identify potential points of interventions unique and in common between profiles. Specifically, we identified unique profiles that may be overlooked or treated as outliers, but may be in dire need of intervention. We also identified possible interactions of social determinants, health, and other substance dependence or abuse unique in each profile that could inform health policy and be a point of intervention to mitigate the distal effects of opioid overdose and death. Furthermore, each profile identified the probability of opioid type that can help develop as well as boost the efficiency and efficacy of interventions and therapies (e.g., medication-assisted treatment). As such, our primary goal was to identify profiles by which interventions can be tailored to noninstitutionalized populations especially from those that may be hidden in survey data.

## 5. Limitations

The NSDUH is a nationally representative instrument for collecting estimates of drug use and mental health. There are, however, limitations associated with using this dataset. One limitation is the use of self-report data, which is subject to the individual bias, truthfulness, and recollection of the responder. To address this issue, the NSDUH employed the use of audio computer-assisted self-interview (ACASI) software instead of human interviewers. While studies have established the validity of the NSDUH, the ACASI design and other implementation procedures are designed to boost recall. Nevertheless, as is the nature with these survey types, a level of under- and over-reporting exists [3]. For the purposes of recall in the 2017 cycle, prescription drug inquiries for specific and related medications allowed participants to report any use/misuse in the past 12 months to allow for the data collection of a given active ingredient. These self-reports do not guarantee accuracy in identifying the drugs taken, particularly when drugs are reported by brand name. Furthermore, the 2017 NSDUH did not include a section for synthetic opioids like fentanyl. Another limitation of the NSDUH is the data are not longitudinal, but cross-sectional. Thus, each survey cycle offers a momentary prevalence of substance use. Finally, although the data are nationally representative, they do exclude a small population subset. The NSDUH targets noninstitutionalized US citizens, so active-duty military members and institutionalized groups (e.g., prisoners, hospital patients, treatment center patients, and nursing home members) are excluded. Therefore, if substance use differs between US noninstitutionalized and institutionalized groups by more than approximately 3%, estimates provided by the NSDUH data may be inaccurate for the total US population [21]. Heroin use prevalence has also been similarly suggested to not be representative as it is a less commonly used drug [3,21]. Finally, due to the cross-sectional nature of the survey data, we cannot ascertain the directionality of heroin or prescription pain reliever initiation.

## 6. Conclusions

Health researchers must first identify and understand opioid misuse risk profiles to prevent use disorders that may lead to adverse outcomes such as overdose and death. Understanding risk is critical to create meaningful prevention strategies and develop targeted interventions, rather than relying on universal blanket interventions that may fail medically underserved or underrepresented groups [9]. Our person-centered approach provided a manner to minimize researcher bias and identify opioid misuse risk profiles in context of co-occurring sociodemographic and health indicators with concurrent substance use informed by current epidemiological studies. Consequently, we were able to identify five misuse risk profiles among US noninstitutionalized adults. Each profile identified provided a possible emergent risk group that should be further examined to determine their levels of increasing or transitioning risk in to use disorder. Of note was that all profiles identified will need tailored intervention programs as all are at possible risk of overdose. As such, each risk profile has unique and common risk factors that can be focused on to create the most efficient and efficacious interventions.

## Figures and Tables

**Table 1:** Sample descriptive characteristics (*n* = 476; Weighted *n* = 2,018,922).

	*n*	%
Age Groups		
18–25	102	21.5
26–34	133	27.9
35–49	111	23.4
50 and older	129	27.2
Sex		
Male	288	60.6
Female	188	39.4
Race/Ethnicity		
Non-Hispanic white	371	77.9
Non-Hispanic black	47	9.8
Hispanic	32	6.7
Other	27	5.6
Sexual Identity		
Heterosexual	421	90.5
Sexual minority	44	9.5
Family Income		
Less than $20,000	131	27.5
$20,000-$49,999	156	32.7
$50,000 or more	190	39.8
Educational Attainment		
Less than high school	86	18.1
High school graduate	135	28.5
Some college/associate’s degree	187	39.2
College graduate	68	14.2
Area of Residence		
CBSA of 1 million or more	225	47.2
CBSA with less than 1 million	231	48.4
Not in a CBSA	21	4.3
Employment (past week)		
Employed	253	53.3
Unemployed	66	13.9
Disabled	65	13.8
Other	90	19.0
Ever Arrested and Booked		
No	188	38.2
Yes	293	61.8
Self-reported Health Status		
Fair/poor	134	28.3
Good	176	37.1
Very good/excellent	164	34.6
Covered by Private Health Insurance		
No	305	64.4
Yes	169	35.6
Serious Psychological Distress (past year)		
No	208	43.8
Yes	268	56.2
Suicidality (past year)		
No	340	71.9
Yes	133	28.1
Nicotine Dependence (past month)		
No	209	44.4
Yes	267	55.6
Alcohol Dependence or Abuse (past year)		
No/Unknown	334	75.3
Yes	142	24.7
Marijuana Dependence or Abuse (past year)		
No/Unknown	394	82.4
Yes	82	17.6
Other Illicit Substance Dependence or Abuse (past year)		
No/Unknown	301	64.8
Yes	60	35.3
Opioid Dependence or Abuse (past year)		
Heroin only	102	21.5
Prescription pain reliever only	314	66.0
Heroin and prescription	59	12.5

Notes. CBSA, core-based statistical area. Latent Class Analysis Model Findings.

**Table 2:** Five-class solution conditional probabilities from latent class analysis (*n* = 476; Weighted *n* = 2,018,922).

	Class 1 Single Heroin or Prescription Misuse	Class 2 Female Prescription Pain Reliever Misuse	Class 3 Younger Polyopioid Misuse	Class 4 Older Polyopioid Misuse	Class 5 Older Male Dual Heroin and/or Prescription Misuse
	27%	20%	38%	10%	5%
	(*n* = 130)	(*n* = 97)	(*n* = 179)	(*n* = 48)	(*n* = 22)
Age group					
18–25 years old	0.218	0.324	0.236	0.000	0.000
26–34 years old	0.202	0.313	0.424	0.000	0.000
35–49 years old	0.229	0.181	0.299	0.231	0.000
50 and older	0.351	0.181	0.041	0.769	1.000
Sex/Gender					
Male	0.630	0.221	0.710	0.790	1.000
Female	0.370	0.779	0.290	0.210	0.000
Race/Ethnicity					
Non-Hispanic white	0.781	0.792	0.763	0.695	1.000
Non-Hispanic black	0.100	0.074	0.085	0.236	0.000
Other race	0.053	0.068	0.055	0.070	0.000
Hispanic	0.065	0.066	0.096	0.000	0.000
Sexual Identity					
Heterosexual	0.940	0.779	0.938	0.926	1.000
Lesbian, gay, or bisexual	0.060	0.221	0.062	0.074	0.000
Family Income					
Less than $20,000	0.000	0.342	0.395	0.529	0.013
$20,000–$49,999	0.282	0.404	0.325	0.344	0.214
$50,000 or more	0.718	0.254	0.280	0.127	0.773
Educational Attainment					
Less than high school	0.060	0.112	0.199	0.514	0.325
High school grad	0.169	0.214	0.456	0.237	0.000
Some college/associate’s	0.472	0.500	0.345	0.250	0.129
College graduate	0.299	0.174	0.000	0.000	0.547
Area of Residence					
CBSA with ≥1 million	0.495	0.435	0.408	0.484	1.000
CBSA with <1 million	0.468	0.528	0.538	0.462	0.000
Not in a CBSA	0.038	0.036	0.054	0.055	0.000
Employment (past week)					
Employed full/part time	0.927	0.449	0.421	0.203	0.347
Unemployed	0.003	0.163	0.274	0.000	0.000
Disabled	0.016	0.087	0.037	0.797	0.440
Other	0.054	0.301	0.268	0.000	0.214
Arrested and booked in lifetime					
No	0.572	0.672	0.168	0.082	0.342
Yes	0.428	0.328	0.832	0.918	0.658
Self-reported health status					
Fair/Poor	0.081	0.414	0.190	1.000	0.000
Good	0.448	0.213	0.430	0.000	1.000
Very good/Excellent	0.472	0.373	0.380	0.000	0.000
Private health insurance					
No	0.000	0.660	0.952	1.000	1.000
Yes	1.000	0.340	0.048	0.000	0.000
Serious psychological distress					
No	0.604	0.104	0.526	0.281	0.667
Yes	0.396	0.896	0.474	0.719	0.333
Suicidality					
No	0.780	0.468	0.839	0.779	0.440
Yes	0.220	0.532	0.161	0.221	0.560
Opioid dependence or abuse					
Heroin only	0.149	0.040	0.371	0.286	0.000
Pain reliever only	0.785	0.835	0.483	0.558	0.786
Heroin and pain reliever	0.066	0.125	0.146	0.156	0.214

Notes. Increasing red coloration corresponds to increasing conditional probability.

**Table 3:** Multivariate logistic regression of substance dependence and abuse covariates on five-class solution model using Class 2 or female prescription misuse profile as reference.

Odds Ratios (95% Confidence Intervals)				
	Class 1 Single heroin or prescription misuse	Class 3 Younger polyopioid misuse	Class 4 Older polyopioid misuse	Class 5 Older male dual heroin and/or prescription misuse
Nicotine Dependence	0.56 (0.24, 1.30)	4.49 (1.94, 10.4) *	3.74 (1.00, 14.0) *	2.17 (0.30, 15.9)
Alcohol Dependence/Abuse	0.56 (0.24, 1.30)	0.41 (0.15, 1.13)	0.39 (0.13, 1.13)	0.37 (0.03, 4.02)
Marijuana Dependence/Abuse	0.63 (0.19, 0.83)	0.22 (0.08, 0.60) *	1.39 (0.45, 4.34)	2.49 (0.24, 25.7)
Other Substance Dependence/Abuse	0.53 (0.18, 1.53)	1.51 (0.63, 3.65)	1.50 (0.53, 4.25)	0.95 (0.16, 5.55)

Note. * = *p <* 0.05.
